# Homoeologous *GSL-ELONG* gene replacement for manipulation of aliphatic glucosinolates in *Brassica rapa* L. by marker assisted selection

**DOI:** 10.3389/fpls.2013.00055

**Published:** 2013-03-25

**Authors:** Arvind H. Hirani, Carla D. Zelmer, Peter B. E. McVetty, Fouad Daayf, Genyi Li

**Affiliations:** The Department of Plant Science, University of ManitobaWinnipeg, MB, Canada.

**Keywords:** *Brassica rapa*, *Brassica oleracea*, homeologous recombination, SCAR markers, MAS, glucosinolates

## Abstract

Aliphatic glucosinolates are the predominant sulfur-rich plant secondary metabolites in economically important *Brassica* crops. Glucosinolates and their hydrolysis products are involved in plant–microbe, plant–insect, plant–animal, and plant–human interactions. It is, therefore, important to manipulate glucosinolate profiles and contents in *Brassica* species. In this study, aliphatic glucosinolates were genetically manipulated through homoeologous recombination in backcross lines followed by marker assisted selection in *B. rapa*. A resynthesized *B. napus* line, from a cross between *B. rapa *and *B. oleracea*, was backcrossed with Chinese cabbage doubled haploid line, RI16. Marker assisted selection for non-functional gene was performed in each backcross generations. Advanced backcross progenies (BC_3_F_2_) were developed to identify homoeologous gene replacement and/or introgression. Reduction in 5C aliphatic glucosinolates (gluconapoleiferin, glucoalyssin, and glucobrassicanapin) was observed in BC_3_F_2 _progenies of the recurrent parent that carried the *GSL-ELONG*^-^ gene. The *GSL-ELONG*^-^ positive backcross progenies were also screened by the A-genome and *BraGSL-ELONG* gene specific marker, which linked with 5C aliphatic glucosinolates. The A-genome specific marker was absent in the plants of advanced backcross progenies which showed reduction in 5C aliphatic glucosinolates. The results suggest that the functional allele had been replaced by the non-functional *GSL-ELONG*^-^ allele from *B. oleracea*. Some advanced backcross progenies (BC_3_F_2_) positive for the* GSL-ELONG*^-^ allele and the A-genome specific SCAR marker BraMAM1-1 did not show reduction in 5C aliphatic glucosinolates, suggesting that *GSL-ELONG*^-^ allele is recessive. Replacement of the functional locus in the A-genome by non-functional counterpart in the C-genome reduced the content of 5C aliphatic glucosinolates in *B. rapa* seeds with 20 μmol/g.

## INTRODUCTION

In the genus* Brassica*, three diploid species, *B. rapa* L., *B. oleracea* L., and *B. nigra* L. Koch, are the evolutionary original genome donors of three amphidiploid species *B. napus* L., *B. juncea* L. Czern. & Coss., and *B. carinata* Br. These *Brassica* species are economically important crops and are cultivated for edible oil, industrial oil, and biodiesel. Since the introduction of *Brassica* species as crops in agricultural systems, many traits related to agronomy, morphology, physiology, yield, disease resistance, and end product quality have been improved. Most trait improvements have been achieved through conventional breeding approaches such as intraspecific and interspecific hybridization followed by selection. The development and analysis of alien chromosome addition lines were initiated in the 1990s for dissecting *Brassica* genomes for gene mapping and genetic analyses. Similarly, chromosome addition lines have also been deployed for the introgression of suitable traits from wild or weedy relatives to the existing cultivars. Alien chromosome addition monosomic and disomic lines have been successfully developed and proven to be useful resources for gene identification and chromosome homoeology studies between *Brassica* genomes of *B. rapa* × *B. oleracea*, *B. napus* × *B. nigra*, and *B. rapa* × *B. oxyrrhina *(McGrath and Quiros, 1990; Hu and Quiros, 1991; Chen et al., 1992; Cheng et al., 1994; Srinivasan et al., 1998). Several agronomic, disease resistance, morphological, and quality traits such as erucic acid content, flowering time, seed coat color, and stem rot resistance have been successfully introgressed into cultivated *Brassica* crops through regular two parental crosses and subsequent backcrossing (Banga, 1988; Cheng et al., 1994; Navabi et al., 2010).

Wide hybridization requires formation of bivalents or multivalents between homoeologous chromosomes for recombination, which is highly affected by the genes that regulate pairing and homoeology of alien and host chromosomes. The mutant *Ph1* gene, meiosis pairing regulator, resulted in synapsis and recombination between homoeologous chromosomes in wheat (Feldman, 1966; Martinez et al., 2001). Using the *Ph1* mutant, several biotic and abiotic stress tolerance genes have been introgressed through interspecific crosses in cultivated wheat (King et al., 1997, Kuraparthy et al., 2009).

On the other hand, Dover and Riley (1972) reported success of wide hybridization in wheat in the presence of a functional *Ph1 *locus. They suggested that several genes from *Aegilops* species suppressed the activity of the *Ph1 *gene during meiosis, which resulted in successful multivalent formation and subsequent homoeologous recombination. Similar meiotic regulatory mechanisms might be involved in interspecific or intergeneric *Brassica* crosses. In *B. napus*, a *PrBn* (*Pairing Regulator in B. napus*) gene located in the C-genome of *B. napus*, is reported to be involved in the regulation of non-homologous cross over during meiosis (Jenczewski et al., 2003; Nicolas et al., 2009). Szadkowski et al. (2010) reported genome blending mechanism in the first meiosis of resynthesized *B. napus* by formation of bivalent and multivalent between homeologous chromosomes. In addition to that, genomic rearrangements including insertion and/or deletion of parental fragments and appearance of novel fragments reported in the second to fifth selfing generation of resynthesized *B. napus* (Song et al., 1995). Besides that, non-reciprocal translocation events are also observed in synthetic *B. napus* by Sharpe et al. (1995). Similarly, Gaeta et al. (2007) reported homeologous non-reciprocal translocations between the A- and C-genome in early and subsequent advanced selfing generation of resynthesized *B. napus*. This suggests genome shuffling phenomenon in resynthesized *B. napus* creates genetic variability due to several recombination events and resulting phenotypic variations benefits breeders to genetically manipulate important traits such as, agronomic, disease resistance, and seed quality traits.

Glucosinolates, a class of nitrogen and sulfur containing plant secondary metabolites are found in *Brassica* species. Glucosinolates and their hydrolysis products are functionally associated with plants, microbes, insects, animals, and humans. Biosynthesis of glucosinolates occurs in three major steps namely, core-structure formation, side chain elongation, and side chain modification. Extensive studies on glucosinolates suggest that multi-gene family regulate these three steps of glucosinolate biosynthesis in the model plant *Arabidopsis thaliana* (Compos de Quiros et al., 2000; Kliebenstein et al., 2001a,c; Textor et al., 2007; Li et al., 2008). In *B. rapa*, *BraGSL-ELONG* gene family ortholog to *AtMAM* genes in *Arabidopsis* controls the side chain elongation steps of glucosinolate biosynthesis. Two genes (*BoGSL-ELONG* and *BoGSL-PRO*) are involved in side chain elongation, and a gene (*BoGSL-ALK*) is involved in side chain modification were identified and characterized in *B. oleracea *(Li and Quiros, 2002, 2003; Gao et al., 2004). Li and Quiros (2002) studied various accessions of *B. oleracea* for glucosinolate content and reported that white cauliflower produces 3C due to a functional *BoGSL-PRO *gene. On the other hand, lack of 4C aliphatic glucosinolates was due to a mutation at the splicing site of intron 3 of* BoGSL-ELONG*, leading to a non-functional gene in white cauliflower. In addition, they also reported that broccoli produced exclusively glucoraphanin, a 4C aliphatic glucosinolate, suggesting that broccoli has a non-functional *BoGSL-ALK* and a functional *BoGSL-ELONG*. Gene specific molecular markers were developed based on glucosinolate biosynthesis genes in *B. oleracea* for marker assisted selection to manipulate quality and quantity of aliphatic glucosinolates (Li and Quiros, 2002, 2003). Most recently, whole genome sequence of *B. rapa* spp. *pekinensis* cv. Chiifu-401-42 is publicly available with annotation of about 41000 genes (Wang et al., 2011, http://www.brassicadb.org). Sequenced genome information has opened an avenue for comparative analysis of genome with *Arabidopsis* for glucosinolate biosynthesis genes. Wang et al. (2011) reported over 100 orthologous genes for various steps of glucosinolate biosynthesis pathway in *B. rapa*, which revealed seven loci for *BraGSL-ELONG* genes for side chain elongation of aliphatic glucosinolates. Development and utilization of gene and/or loci specific molecular markers using sequenced genome would hasten marker assisted selection for improving quality traits like glucosinolates in *Brassica* vegetables and oilseeds.

In this study, we performed genetic manipulation of aliphatic glucosinolate profile and content in *B. rapa* through homoeologous gene replacement from *B. oleracea* (white cauliflower) using marker assisted selection. In this study, resynthesized *B. napus* lines were developed through interspecific hybridization between *B. rapa* and *B. oleracea*. Synthetic *B. napus* was backcrossed to *B. rapa* to develop *B. rapa-B. oleracea* chromosome addition lines harboring *B. oleracea* non-functional glucosinolate gene, *GSL-ELONG*^-^ through marker assisted selection. The *Brassica *A-genome possesses at least four loci of the *BraGSL-ELONG* gene involved in 4C and 5C aliphatic glucosinolate biosynthesis (Bisht et al., 2009). Based on this knowledge, marker assisted backcross breeding was performed to replace the functional *BraGSL-ELONG*^+^ locus/loci of *B. rapa* with the non-functional *GSL-ELONG*^-^ allele from *B. oleracea* to manipulate 4C and/or 5C aliphatic glucosinolates in this A-genome species. The advanced backcross families with the *GSL-ELONG*^-^ gene were identified with gene specific markers and evaluated for glucosinolate profile and content.

## MATERIALS AND METHODS

### PLANT MATERIALS AND BACKCROSS BREEDING SCHEME

**FIGURE 1:**
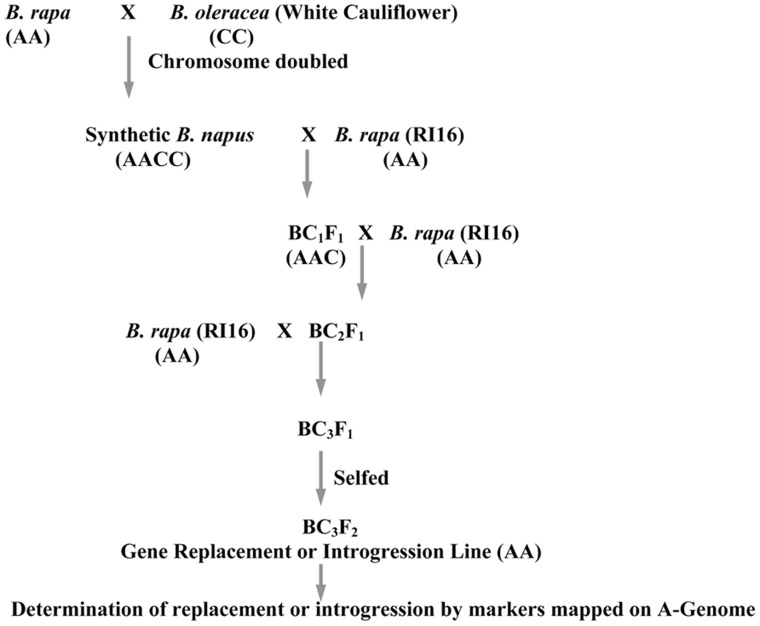
**Schematic diagram for introgression or replacement of glucosinolate genes in *B. rapa* from *B. oleracea***.

A high glucosinolate content *B. rapa* double haploid (DH) line, RI16 was crossed with a *B. oleracea* white cauliflower accession Snowball 76, and an embryo rescue technique was employed to produce resynthesized *B. napus* lines. The Chinese cabbage DH line, RI16 was derived from a hybrid cultivar, Summer Light 50 (Xiayang originally from Japan). One resynthesized *B. napus* line was backcrossed with *B. rapa* recurrent parent, Chinese cabbage (RI16) to produce backcross progenies. During recurrent backcrossing, marker assisted selection was performed in each generation with selection for genes of interest in the C-genome. In the first and second backcross cycle, recurrent parents were used as male parents. Additional reciprocal backcross was performed to enhance allosyndetical homoeologous recombination between the A-genome and alien chromosomes of *B. oleracea*. Advanced backcross progenies (BC_3_F_1_) were selfed to produce offspring homozygous for the replaced major glucosinolate gene *GSL-ELONG*^-^ (**Figure 1**).

### DNA EXTRACTION AND C-GENOME SPECIFIC SCAR MARKER ASSISTED SELECTION

For screening and selection, DNA was extracted from leaf tissues of 2 week old seedlings using a modified cetyltrimethylammonium bromide (CTAB) method described by Li and Quiros (2001). Instead of individual samples in Eppendorf tubes, 96 deep well plates were used. About 0.1 g leaf tissues from individual plants were placed in the wells and ground in liquid nitrogen. 500 μl 2× CTAB buffer (2% CTAB, 20 mM EDTA, 100 mM Tris, 1.4 M NaCl, pH 8.0) was added to each well and then samples were incubated at 65°C for 1.5 h. Subsequently, 400 μl chloroform was added to each well followed by vigorous mixing and centrifugation at 6200 rpm for 10 min. Ninety microliters of the supernatant was transferred to a 96-well plate and then DNA was precipitated with 50 μl iso-propanol followed by centrifugation at 6200 rpm for 5 min. The DNA pellet was washed with 70% ethanol, air dried and then dissolved in 80 μl dH_2_O.

Marker assisted selection of non-functional *BoGSL-ELONG* (*GSL-ELONG*^-^) gene in backcross progenies were performed using the C-genome specific SCAR markers. The polymerase chain reaction (PCR) was conducted using the primer pairs PM25 + PM13 and IPM8 + IPM7 for *GSL-ELONG*^-^(**Table 1**). Each PCR reaction of 10 μl consisted of 7.0 μl dH_2_O, 1.0 μl 10× PCR buffer, 0.3 μl 50 mM MgCl_2_, 0.15 μl 25 mM deoxyribonucleotide triphosphate (dNTP), 0.1 μl *Taq* polymerases, 0.15 μl 10 μM forward and reverse primers and 2.5 μl template DNA. The PCR program was 94°C for the initial 4 min to denature genomic DNA followed by 35 cycles of 94°C for 1 min, 55°C for 1 min and 72°C for 1.5 min. For all backcross generations, the *GSL-ELONG*^-^ gene specific SCAR markers were scored on 1.2% agarose gels. One to seven plants from each family were selected using SCAR markers for the next backcross with the recurrent parents.

**Table 1:** List of primers used for backcross breeding and their sequence information and genome specificity.

Gene (marker)	Primers	Sequence 5′–3′	Specificity
*GSL-ELONG*^-^	PM25	CCTGTGAGACGTTAATACC	C-genome
(PM25 + PM13)	PM13	GAGCTTGAGTTCTATACGC	
*GSL-ELONG*^-^	IPM8	CTTCGAGTAGACATCATGGA	C-genome
(IPM8 + IPM7)	IPM7	AAAGCTATTGTGCGAGGAC	
*GSL-ELONG*^+^	Br-MAM1-1F	GTTTCCCTGCGTCATCAGA	A-genome
(BraMAM1-1)	Br-MAM1-1R	CTAAGCTCTTCGCATAGCTA	
*GSL-ELONG*^+^	AE2F	CAGTCAAATTTACCGCCTT	A-genome
(BraAE2)	AE2R	GGTGGCTTTCGCGGACAC	

### LEAF AND SEED GLUCOSINOLATE EXTRACTION

Leaf glucosinolate was extracted from early (BC_1_F_1_) and advanced (BC_3_F_1_) backcross progenies to determine glucosinolate profiles. Glucosinolate analysis was performed on BC_3_F_2_ seeds from the plants positive for the C-genome SCAR markers. Total glucosinolate content was extracted from 200 mg air dried seeds and 250 mg fresh young leaf tissues. Leaf and seed glucosinolate purification and overnight desulfation reactions were performed using sephadex (Sigma-Aldrish, Canada) and purified sulfatase from *Helix pomatia* (Sigma-Aldrish, Canada) as described by Kliebenstein et al. (2001b) with some minor modifications. Final desulfoglucosinolates were eluted into 400 μl distilled water: 70% methanol (1:1 v/v).

### DETECTION AND QUANTIFICATION OF ALIPHATIC GLUCOSINOLATES IN BACKCROSS PROGENIES

Leaf and seed desulfoglucosinolate separation and quantification was performed in a 5-μm column (LichroCART® 250-4 RP18, Fisher Scientific, Ottawa, Canada) coupled with the Alliance® reverse phase high-performance liquid chromatography (HPLC; Waters 2695) and photodiode array detector (Waters 996) system (Waters, MA, USA). Desulfoglucosinolates were separated using mobile phase HPLC grade methanol (A) and distilled water (B) at a flow rate of 1 ml/min. Both solvents were set up at gradients of an 8-min 7:93 A/B (v/v), a 4-min 15:85 A/B (v/v), an 18-min 55:45 A/B (v/v), a 5-min 92:8 A/B (v/v), a 5-min 92:8 A/B (v/v), a 5-min 1.5:98.5 A/B (v/v), a 3-min 1.5:98.5 A/B (v/v), and final 4-min 0:100 A/B (v/v) with total running time of 52 min. Individual glucosinolates were identified according to retention times and quantity was calculated in micromoles per gram and adjusted with relative response factors as described by Brown et al.(2003; **Table 2**).

**Table 2:** List of glucosinolates with their trivial and chemical names and numbers of side chain carbons.

Trivial name	Peak no.	Chemical name	#C in side chain
Progoitrin (Pro)	1	2-Hydroxybutenyl	4C
Sinigrin (Sin)	2	2-Propenyl	3C
Gluconapoleiferin (Gnapol)	3	2-Hydroxy-4-pentenyl	5C
Glucoalyssin (Galy)	4	5-Methylsulfinylpentyl	5C
Gluconapin (Gnap)	5	3-Butenyl	4C
4-Hydorxyglucobrassicin (4OH)	6	4-Hydorxyglucobrassicin	Indole
4-Methoxyglucobrassicin (4MGB)	7	4-Methoxyglucobrassicin	Indole
Glucobrassicanapin (Gbnap)	8	4-Pentenyl	5C
Total alipahtic GSL (Tagsl)	–	Sum of aliphatic GSL	3C + 4C + 5C

### DETERMINATION OF GENE REPLACEMENT THROUGH THE A-GENOME SPECIFIC MARKERS

The A-genome and *BraGSL-ELONG* gene specific SCAR markers (**Table 1**) for the glucosinolate side chain elongation were used to determine replacement of major effect locus/loci in *B. rapa*. Advanced backcross progenies were screened with the A-genome specific BraMAM1-1 and BraAE2 SCAR markers. These SCAR markers are dominant markers; therefore, absence of band was considered evidence of putative replacement/transposition of the native allele(s) with the corresponding allele from the C-genome.

### CYTOLOGICAL ANALYSIS OF CHROMOSOMES

Chromosomes were counts in BC_3_F_3_ plants from those lines which reduced 5C aliphatic glucosinolates. Flower buds were fixed for 24 h in propionic acid : absolute ethanol (1:3 v/v) and ferric chloride (~0.03%) was added as a mordant. Subsequently, buds were rinsed and stored in 70% ethanol. Flower buds were dissected and anthers were squashed in a drop of 1% acetocarmine on a glass slide. For chromosome counting, 10–20 pollen mother cells (PMCs) per flower bud, at least one bud per plant and two to four plants per lines were examined.

## RESULTS

### CROSSABILITY OF DIGENOMIC TRIPLOID HYBRID WITH RECURRENT *B. rapa*

A resynthesized *B. napus* line was developed by a cross between *B. rapa* genotype (high glucosinolate content) and *B. oleracea* white cauliflower genotype (lack of 4C and 5C aliphatic glucosinolate content). The resynthesized *B. napus* line was backcrossed with *B. rapa* DH line RI16 of Chinese cabbage. The recurrent parent Chinese cabbage had distinct glucosinolate profile and content. The Chinese cabbage line produced gluconapin and trace amounts of progoitrin as 4C glucosinolates together with glucoalyssin and glucobrassicanapin as 5C aliphatic glucosinolates. Crossing of the resynthesized *B. napus* line with the *B. rapa* recurrent parent, RI16, on average, produced four seeds per silique (2–8), and resulted in digenomic triploid hybrid (AAC) plants. These AAC hybrid plants were subsequently backcrossed with the same *B. rapa* recurrent parent. In the case of the second backcross (BC_2_F_1_), on average, three seeds per silique (0–5) were recorded. Seed setting in BC_2_ hybrids were very low, about 40% silique did not produce seeds, apparently due to genomic irregularities in the plants. Positive plants (BC_2_F_1_) for the C-genome specific SCAR markers were selected and reciprocally backcrossed to the RI16 to produce BC_3_F_1_ seeds. In BC_3_F_1_ progenies, normal seed setting was observed, it could be due to reciprocal cross conducted that recovered recurrent genome and eliminated extra chromosomes from the genome. The C-genome specific SCAR marker positive plants (30%) were selfed to produce homozygous progenies (BC_3_F_2_). Seed setting was normal in all the homozygous progenies of BC_3_F_1_.

### GENOME SPECIFIC SCAR MARKERS FOR ALIPHATIC GLUCOSINOLATES

**FIGURE 2:**
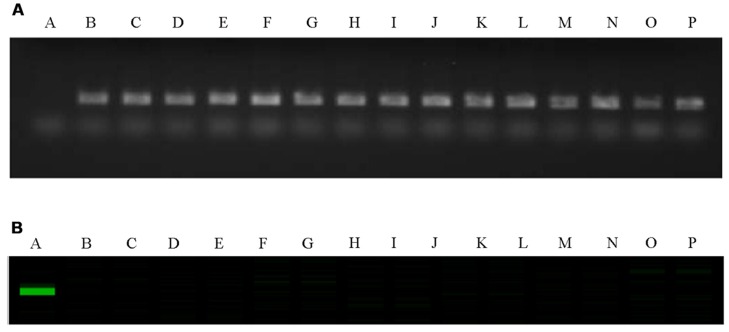
**Polymerase chain reaction (PCR) gel image of *GSL-ELONG*^-^/*GSL-ELONG*^+^ locus, in the parental lines RI16, white cauliflower and selected backcross progenies of BC_3_F_2_**. A-RI16 (*B. rapa*), B-white cauliflower (*B. oleracea*), C to P- selected backcross progenies of BC_3_F_2_. **(A)** C-genome and *GSL-ELONG*^-^ gene specific SCAR marker, PM25 + PM13; **(B)** A-genome and *GSL-ELONG*^+^ gene specific SCAR marker, BraMAM1-1.

The C-genome specific SCAR markers PM25 + PM13 and IPM8 + IPM7 were used to screen for *GSL-ELONG*^-^ gene in marker assisted selection of backcross progenies (**Figure 2**). All backcross generations (BC_1_F_1_ to BC_3_F_2_) were screened with gene specific SCAR markers to obtain positive plants (**Table 3**). Marker positive plants in BC_1_F_1_ to BC_3_F_1_ could be due to the presence of the C-genome addition chromosome or due to the replacement or introgression of a C-genome fragment containing the *GSL-ELONG*^-^ gene into the A-genome. In the backcross generations, loss of both the C-genome specific SCAR markers (PM25 + PM13 and IPM8 + IPM7) was observed in BC_1_ (six families), BC_2_ (four families), and BC_3_ (six families). The loss of these markers could be due to disappearance of additional chromosome without introgression and/or replacement events. On the other hand, four families in BC_1_, three families in BC_2_, and four families in BC_3_ were detected with the C-genome specific markers for *GSL-ELONG*^-^ gene. This suggests that homoeologous recombination events might had occurred and resulted in replacement or introgression of the chromosomal fragments of the C-genome into the A-genome. There are three possibilities for the C-genome specific SCAR marker behavior, (i) replacement with the A-genome fragment, (ii) introgression of the C-genome fragment into the A-genome, and (iii) existence of additional chromosome in the backcross progenies (aneuploid). The A-genome specific SCAR markers for *GSL-ELONG*^+^ loci of chromosome A3 (BraMAM1-1) and chromosome A2 (BraAE2) were used to screen the BC_3_F_2_ population of the recurrent parent RI16 to investigate the occurrence of gene replacement or introgression. Fifteen BC_3_F_2_ plants of this population did not show the A-genome specific marker BraMAM1-1 for *GSL-ELONG*^+^ locus of chromosome A3, suggesting that homoeologous recombination and gene replacement had occurred. On the other hand, eight BC_3_F_2_ families displayed both the A- and C-genome specific markers, suggesting that introgression of *GSL-ELONG*^-^ in the A-genome had occurred. However, no phenotypic changes in leaf and seed glucosinolates were observed in these families.

**Table 3:** Aliphatic glucosinolate contents in parental lines *B. rapa* (RI16), *B. oleracea* (Snowball 76), and synthetic *B. napus* (RI16 × Snowball 76).

Genotypes	Pro	Sin	Gnapol	Galy	Gnap	Gbnap	Sum4C	Sum5C	Tagsl
*B. rapa* (RI16)	0.65	1.74	0.32	2.73	36.21	15.45	37.02	18.51	55.52
*B. oleracea* (Snowball 76)	1.31	72.51	0.00	0.00	0.00	0.25	1.31	0.25	1.56
*B. napus *(synthetic line)	24.57	0.70	0.85	1.36	27.39	5.10	51.96	7.31	59.27

### MODIFICATION OF ALIPHATIC GLUCOSINOLATE PROFILES IN BACKCROSS PROGENIES

Two parental lines, RI16 (*B. rapa*) containing 37 μmol/g seed 4C and 19 μmol/g seed 5C aliphatic glucosinolates and Snowball 76 (*B. oleracea*) containing 72 μmol/g seed 3C and trance amount of 4C and 5C aliphatic glucosinolates were used to develop resynthesized *B. napus* line containing 52 μmol/g seed 4C and 8 μmol/g seed 5C aliphatic glucosinolates. Total aliphatic glucosinolate contents in seeds of *B. rapa* (RI16), *B. oleracea* (Snowball 76) and resynthesized *B. napus* line were 56, 74, and 60 μmol/g, respectively (**Table 4**).

**Table 4:** *GSL-ELONG*^-^ (PM25 + PM13 and IPM8 + IPM7) marker transmission frequency in interspecific backcross progenies^§^.

Generation	Family name	Total plants	Plants with markers	Plant without markers	Expected plants	MTR (%)
BC_1_F_1_	A	43	8	35	21.5	18.6
	B	30	11	19	15	36.7
	C	28	10	18	14	35.7
	D	23	1	22	11.5	4.3
	E	8	3	5	4	37.5
	F	5	1	4	2.5	20.0
	G	3	-	3	1.5	-
	H	2	1	1	1	50.0
	I	2	1	1	1	50.0
	J	5	-	5	2.5	-
		149	36	113	74.5	24.2
BC_2_F_1_	B01xR	5	2	3	2.5	40.0
	B19xR	6	2	4	3	33.3
	B30xR	13	1	12	6.5	7.7
	C12xR	16	10	6	8	62.5
	C27xR	48	9	39	24	18.8
	H01xR	8	3	5	4	37.5
	I01xR	9	4	5	4.5	44.4
		105	31	74	52.5	29.
BC_3_F_1_	RxC27(28)	38	2	36	19	5.3
	RxB01(2)	6	3	3	3	50.0
	RxC12(1)	6	2	4	3	33.3
	RxC27(23)	23	1	22	11.5	4.3
	RxC27(20)	24	9	15	12	37.5
	RxB03(2)	5	1	4	2.5	20.0
	RxC27(17)	7	3	4	3.5	42.9
	RxC27(2)	2	1	1	1	50.0
	RxC27(5)	24	6	18	12	25.0
	RxC27(10)	6	2	4	3	33.3
		141	30	111	70.5	21.3
BC_3_F_2_	RxB01(2)(3)	48	20	28	36	41.7
	RxB03(2)(4)	48	18	30	36	37.5
	RxC12(1)(6)	24	19	5	18	79.2
	RxC27(20)(1)	48	26	22	36	54.2
	RxC27(20)(10)	48	23	25	36	47.9
	RxC27(20)(17)	24	10	14	18	41.7
	RxC27(20)(18)	24	9	15	18	37.5
	RxC27(20)(2)	24	15	9	18	62.5
	RxC27(20)(23)	48	20	28	36	41.7
	RxC27(20)(5)	24	12	12	18	50.0
	RxC27(28)(14)	24	8	16	18	33.3
	RxC27(28)(2)	24	11	13	18	45.8
	RxC27(28)(3)	24	15	9	18	62.5
		432	206	226	324	47.7

§ Resynthesized *B. napus* × *B. rapa* hybrid, recurrently backcrossed to *B. rapa*; R-RI16. Those backcross lines, which lost C-genome specific marker has not proceeded to next generation.

**FIGURE 3:**
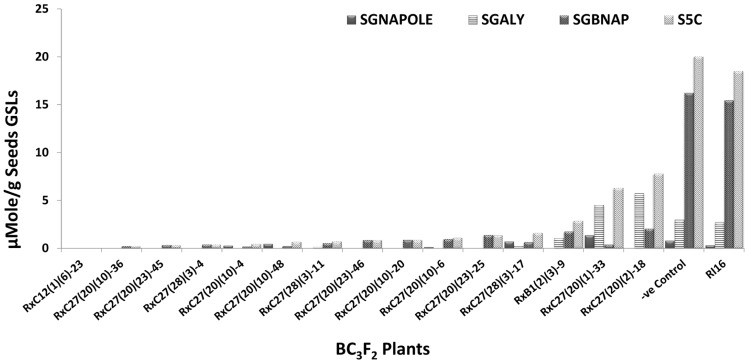
**BC_3_F_2_ plants of RI16 recurrent parent with reduction in 5C aliphatic glucosinolates in seeds**. SGNAPOLE, seed gluconapoleiferin; SGALY, seed glucoalyssin; SGBNAP, seed glucobrassicanapin; S5C, seed sum of aliphatic 5C glucosinolates.

**FIGURE 4:**
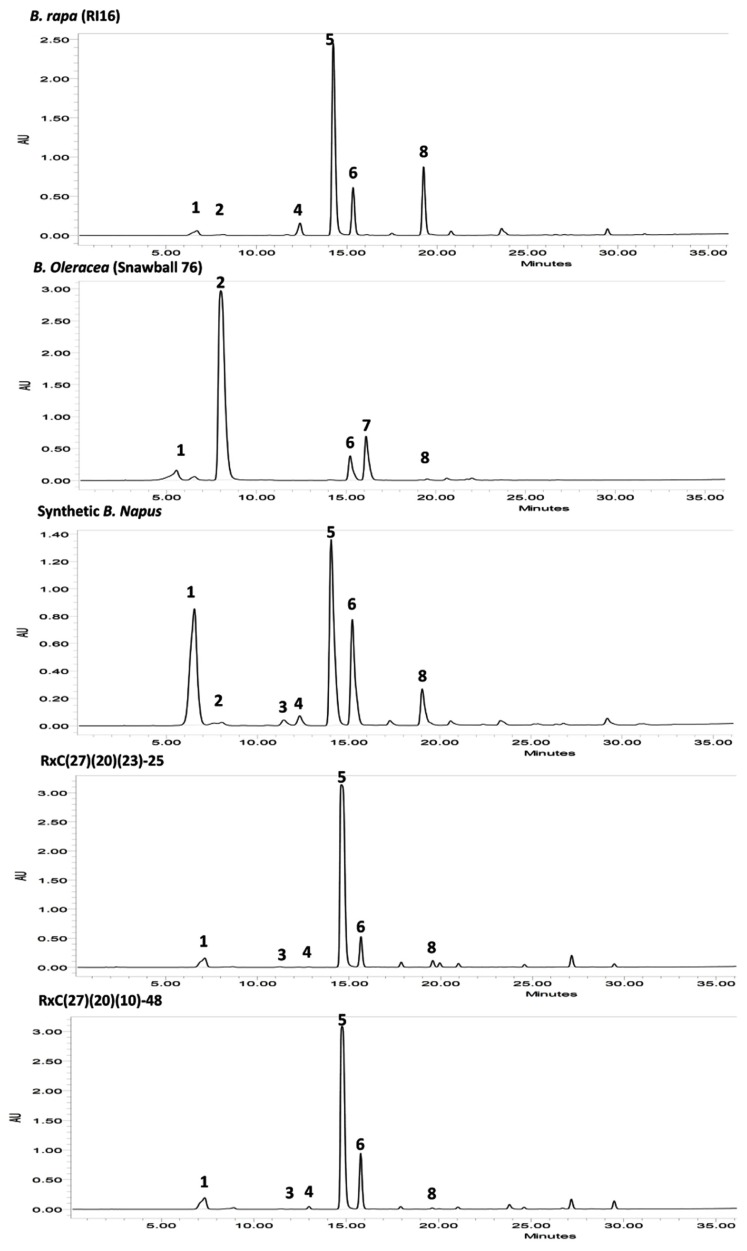
**High-performance liquid chromatography (HPLC) profiles of seed glucosinolates in parental lines *B. rapa* (RI16), *B. oleracea* (Snowball 76), resynthesized *B. napus*, and advanced backcross progenies, RxC27(20)(23)-25 and RxC27(20)(10)-48; *Peak* 1 Progoitrin, *Peak* 2 Sinigrin, *Peak* 3 Gluconapoleiferin, *Peak* 4 Glucoalyssin, *Peak* 5 Gluconapin, *Peak* 6 4-Hydroxyglucobrassicin, *Peak* 7 4-Methoxyglucobrassicin, *Peak* 8 Glucobrassicanapin.** HPLC profiles of advanced backcross plants, RxC27(20)(23)-25 and RxC27(20)(10)-48 displayed reduction in 5C aliphatic glucosinolates, gluconapoleiferin, glucoalyssin, and glucobrassicanapin.

**FIGURE 5:**
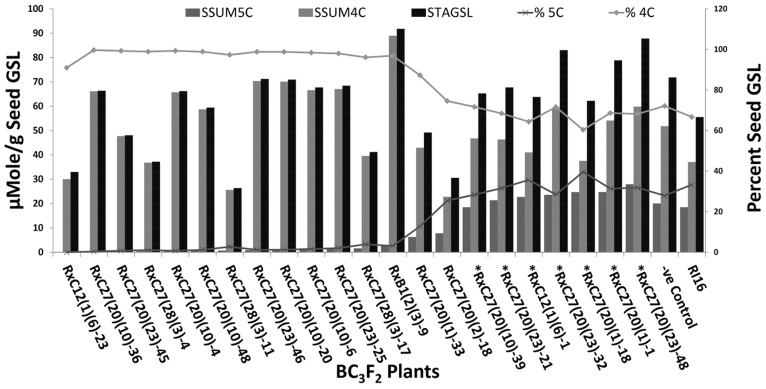
**Percent seed total aliphatic glucosinolates (STGSL), seed sum of 4C GSL (SSUM4C), and seed sum of 5C GSL (SSUM5C) in BC_3_F_2_ plants of RI16 recurrent parent.** Asterick “*” represents BC_3_F_2_ plants with both SCAR markers specific to the A and C-genome. –Ve Control represents BC_3_F_2_ plant with A-genome specific and without C-genome specific SCAR maker.

Fifty BC_3_F_2_ plants positive for *GSL-ELONG*^-^ markers were selected for glucosinolates analysis. A total 15 plants (30%) were found to have reduced 5C aliphatic glucosinolates by five times to their recurrent parent RI16n and all these plants did not show the A-genome specific marker BraMAM1-1 corresponding to *GSL-ELONG*^+^ locus responsible for the biosynthesis of 5C aliphatic glucosinolates (**Figures 3** and **4**). On the other hand, 35 plants (70%) which were positive for the *GSL-ELONG*^-^ marker did not show any changes in glucosinolate profiles due to the existence of corresponding functional alleles of the A-genome. The recurrent parent Chinese cabbage RI16 produced 20 μmole/g seed of 5C aliphatic glucosinolates, whereas some of the backcross progenies of RI16 produced trace amount (<5 μmol/g seed) of 5C aliphatic glucosinolates (**Figures 3** and **4**). The backcross progenies, which reduced 5C aliphatic glucosinolates were increased 4C aliphatic glucosinolates, resulting 4C glucosinolates represent total aliphatic glucosinolates in those progenies (**Figure 5**). Results of the A- and C-genome specific molecular markers and modification of glucosinolate profiles indicate possible functional gene replacement by non-functional alleles.

**FIGURE 6:**
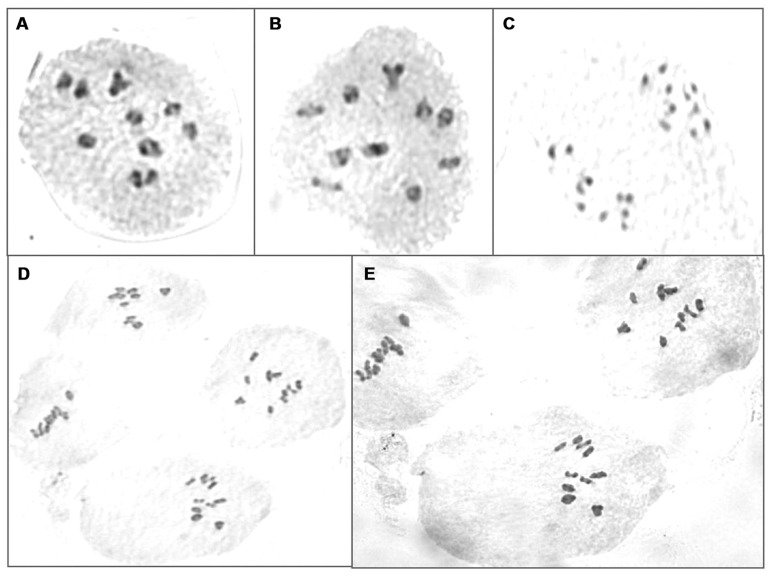
**Chromosome counting of PMCs in BC_3_F_3_ plants which reduced 5C aliphatic glucosinolates**. **(A,B)** Meiotic division at late prephase I in lines RxC27(20)(10)-48 and RxC27(28)(3)-11, respectively; **(C)** telophase I of meiotic division in line RxC27(20)(2)-18; **(D,E)** metaphase I of meiotic division in lines RxC27(20)(23)-25 and RxC12(1)(6)-23, respectively.

Further to validate molecular marker results, chromosomes were counted in BC_3_F_3_ plants which reduced 5C aliphatic glucosinolates. Two to four plants from each BC_3_F_2_ lines were analyzed for chromosome numbers. All PMCs showed normal cell divisions during meiosis and did not show any univalent or lagging chromosomes suggesting that all plants contained 20 chromosomes (**Figure 6**).

## DISCUSSION

Glucosinolate composition and quantity are genetically regulated quantitative traits in *Arabidopsis* and *Brassica* species. *B. rapa* has a high level of genome duplication, which results in more loci with various functional properties (Punjabi et al., 2008). Genome duplications and rearrangements in *B. rapa* have led to a very complex genetic basis for glucosinolate biosynthesis. *B. rapa* and *B. oleracea* diverged quite recently (approximately 7.9 MYA) from a common ancestor when compared to *B. nigra* (approximately between 16 and 18 MYA), therefore, relatively high homoeology exists between the A- and C-genomes (Parkin et al., 1995, 2005; Wroblewski et al., 2000; Punjabi et al., 2008). High homoeology between the A- and C-genomes permits crossing over and recombination events in the crosses of the A- and C-genome holding *Brassica* species.

Chromosomal pairing and recombination between homoeologous chromosomes in digenomic triploid (AAC) lines of *B. rapa * ×* B. oleracea* occurs (Attia and Röbbelen, 1986, 1987), however, a very low rate of homoeologous recombination was also reported (Chen et al., 1992; Mikkelsen et al., 1996; Leflon et al., 2006). Homoeologous recombination and alien chromosome transmission rates depend on chromosome identity and meiotic behavior, resulting in varied alien chromosome transmission rates in different digenomic hybrids (Leflon et al., 2006). In this study, varied *GSL-ELONG*^-^ marker transmission rates (4–79%) were observed in backcross progenies, this suggested variable homoeologous recombination events. This was in accordance with the previous studies reported in *Brassica* and other crop species. Heneen and Jørgensen (2001) reported variable random amplified polymorphic DNA (RAPD) marker transmission frequencies in aneuploid progenies of *B. rapa* × *B. alboglabra*. Kaneko et al. (2003) reported variable marker transmission rates ranging from 26 to 44% in three consecutive generations of *Raphanus sativus* possessing monosomic additional chromosomes of *B. rapa*. Similarly, Shigyo et al. (2003) reported 9– 49% transmission rates of alien chromosomes in eight *Allium* monosomic addition lines. Ali et al. (2001) reported transmission rates from 0 to 32% for chromosome 9 and from 14 to 88% for chromosome 6 of monosomic tomato chromosome addition lines in the cultivated potato. This suggests that different alien chromosomes have very substantial transmission rates in different crop species. Transmission rates appear to be higher through female parents and for the larger chromosomes (Garriga-Calderé et al., 1998).

Functional gene-based trait introgression has been reported in many studies of crosses between *B. napus*, *B. oleracea*, or *B. alboglabra* with *B. rapa* (Guo et al., 1990; Cheng et al., 1994; Heath et al., 1994). However, in *B. rapa*, this is the first report of homoeologous recombination and gene replacement of a functional locus in the A-genome by a non-functional C-genome specific allele. Gene replacement has led to reduction of 5C aliphatic glucosinolates in advanced backcross progenies. Replacement of large or small effect loci might have variable contributions on individual and total aliphatic glucosinolate content due to the existence of multiple loci for the *BraGSL-ELONG* gene in the A-genome. Four and three *BraGSL-ELONG* loci were integrated for glucosinolate side chain elongation gene by Bisht et al. (2009) in the A-genome of *B. juncea* and by Geng et al. (unpublished) in *B. rapa*, respectively. In addition, comparative sequence analysis of *Arabidopsis *glucosinolate biosynthesis genes with the whole genome sequence dataset of *B. rapa* ssp. *pekinensis* cv. chiifu, Wang et al. (2011) revealed three loci for BraMAM1 and four loci for BraMAM3 for side chain elongation genes (BraGSL-ELONG). This suggests existence of genetic complexity for glucosinolate side chain elongation pathway in *B. rapa*. Availability of genome and gene specific molecular markers with known gene function for glucosinolate biosynthesis pathway, breeding would be enhanced for specific glucosinolate profiles and contents in rapeseed and vegetable *Brassica* species. In the quantitative trait locus (QTL) mapping study for glucosinolates in *B. rapa*, Geng et al. (unpublished) identified a single major QTL for 5C aliphatic glucosinolates on linkage group A3 linked with the SCAR marker, BraMAM1-1. Our results are coherent with those findings and indicate that absence of BraMAM1-1 marker in the A-genome lack 5C aliphatic glucosinolates. This suggests that a major *BraGSL-ELONG* locus which elongates glucosinolate side chain for 5C aliphatic glucosinolate biosynthesis is located on chromosome A3 in the A-genome of *B. rapa*. On the other hand, no QTL detected on linkage group A9 suggested that *BraGSL-ELONG* locus on A9 may not play an important role in the biosynthesis of 5C aliphatic glucosinolates in the Chinese cabbage DH line, RI16 used in this study.

Glucosinolate profiles of 15 progenies which reduced 5C aliphatic glucosinolates also displayed increased in 4C aliphatic glucosinolates (**Figure 5**). Similar glucosinolate contents were reported by Liu et al. (2010), 2012 through silencing *BnGSL-ELONG* and *Bn-GSL-ALK* gene families in *B. napus*. These results suggested lack of functional genes at in some steps of chain reactions results in the accumulation of the precursors of glucosinolates in the biosynthesis pathway. For example, in side chain elongation, lack of functional *BraGSL-ELONG* locus/loci for 5C or 4C aliphatic glucosinolates would accumulate 4C or 3C aliphatic glucosinolates. In this study, other *BraGSL-ELONG* loci might have some roles in the biosynthesis of aliphatic glucosinolates including 5C aliphatic glucosinolates, which had not been replaced and resulted in variation of total aliphatic glucosinolate contents in different backcross families. The backcross progenies with random introgression of non-functional allele did not change aliphatic glucosinolate content.

Loss of the markers for the alien chromosome or chromosomal fragment was observed in backcross progenies for *GSL-ELONG*^-^ gene. This loss might be due to the random inclusion of the alien chromosome into the gametes during meiosis. Retention of the marker would then depend on the specific gametes involved during fertilization of backcrosses. Findings also reported loss of markers for QTL or genes in marker assisted backcross selection in crop species (Sebolt et al., 2000; Ramchiary et al., 2007; Szadkowski et al., 2010). In advanced backcross progenies, significance segregation distortion for *GSL-ELONG*^-^ markers was observed. Similar studies reported for marker distortion to non-Mendelian segregation in *Brassica* and wheat (Hu and Quiros, 1991; Ceoloni et al., 1996; Marais et al., 2010; Niu et al., 2011). This is a common feature associated in the breeding with most genetic background carrying alien chromosome.

Replacement or introgression of the *GSL-ELONG*^-^ locus in these backcross progenies would be confirmed by additional backcrossing and sequencing the genomic regions of the replaced genes. Alternatively, resynthesized *B. napus* line would be developed through a cross between a *B. rapa* line with trace amount of 5C GSL and *B. oleracea* white cauliflower which lacks 4C and 5C aliphatic glucosinolates. The Chinese cabbage lines with reduced 5C aliphatic glucosinolates achieved in this study could be used for manipulation of glucosinolate profiles in *B. napus*, *B. juncea*, and other *B. rapa* accessions.

## Conflict of Interest Statement

The authors declare that the research was conducted in the absence of any commercial or financial relationships that could be construed as a potential conflict of interest.
